# Effect of Exogenous Calcium on Tolerance of Winter Wheat to Cold Stress during Stem Elongation Stage

**DOI:** 10.3390/plants12213784

**Published:** 2023-11-06

**Authors:** Maguje Masa Malko, Xinyue Peng, Xing Gao, Jian Cai, Qin Zhou, Xiao Wang, Dong Jiang

**Affiliations:** 1 National Technique Innovation Center for Regional Wheat Production, Key Laboratory of Crop Ecophysiology, Ministry of Agriculture, Nanjing Agricultural University, Nanjing 210095, China; maguje700@gmail.com (M.M.M.); 2019101026@njau.edu.cn (X.P.); 2022101024@njau.edu.cn (X.G.); caijian@njau.edu.cn (J.C.); qinzhou@njau.edu.cn (Q.Z.); jiangd@njau.edu.cn (D.J.); 2 Department of Plant Science, College of Agriculture, Wolaita Sodo University, Wolaita Sodo P.O. Box 138, Ethiopia

**Keywords:** calcium, cold-responsive genes, low-temperature stress, priming, wheat

## Abstract

Low-temperature stress during stem elongation is a major factor limiting wheat yield. While calcium (Ca^2+^) is known to enhance stress tolerance, it’s potential as an alternative to cold priming and the underlying mechanisms in wheat remains unclear. The current study assessed the effects of exogenous Ca^2+^ and calcium inhibitors on wheat growth and related physiology mechanisms under low-temperature stress. The results revealed that exogenous Ca^2+^ increased photosynthesis and antioxidant capacity, lowered cell membrane damage, and ultimately enhanced tolerance to low-temperature stress during the stem elongation stage, compared with the non-exogenous Ca^2+^ treatment. Moreover, exogenous Ca^2+^ induced endogenous Ca^2+^ content and triggered the upregulation of Ca^2+^ signaling and cold-responsive related genes. This study highlights the significance of exogenous Ca^2+^ in enhancing stress tolerance and contributing to wheat yield improvement under low-temperature stress.

## 1. Introduction

Wheat (*Triticum aestivum* L.) is one of the world’s three major grain crops and serves as a staple food for more than 60% of the world’s population. Therefore, ensuring a stable and high wheat yield is essential for global food security [1]. However, with global climate change, extreme cold stress events, in terms of frequency, intensity, and duration, are increasing, resulting in disastrous impacts on agricultural production [2]. Although wheat is a low-temperature tolerant crop with a certain level of cold stress tolerance, it is highly sensitive to low-temperature stress during its stem elongation stage, which results in a decline in grain yield by 5–14% [3]. Low-temperature stress causes disruption of various physiological pathways, photosynthetic apparatus damage, cellular membrane peroxidation, lipid composition changes, and cytoskeleton arrangement changes, eventually affecting plant growth, development, and survival [4]. Thus, improving the low-temperature tolerance of wheat has been recognized as a crucial strategy to ensure stable and sustainable wheat production.

Calcium serves as the second messenger in eukaryotic signal transduction and is involved in signaling pathways that respond to various abiotic stresses including low-temperature stress [5]. In plants, there are a significant number of proteins that can bind to Ca^2+^, referred to as Ca^2+^ receptors. These Ca^2+^ receptors include calcium-dependent protein kinases (CDPKs), calmodulin (CaM), calmodulin-like proteins (CMLs), and calcineurin B-like proteins (CBLs) [6]. These proteins decode the calcium signal, transmit it downstream, and regulate the amplification of cascades, ultimately leading to stimulus-specific physiological responses [7]. Previous studies have indicated that, after the plant perceives cold signals, the expression of genes of transcription factors, i.e., CBFs, is rapidly induced. The CBF protein then activates the cold-responsive gene by binding to the specific cis-elements of the downstream cold-responsive gene COR, thereby enhancing the plant’s cold resistance capabilities [8,9]. The mitogen-activated protein kinase (MAPK) cascade also plays a critical role in eukaryotic cells’ response to various external signals and regulates complex intracellular changes [10].

A previous study demonstrated that exogenous Ca^2+^ can alleviate leaf damage and growth inhibition under cold stress in peanuts [11]. In this regard, spraying Ca^2+^ on the peanut leaves could protect the photosystem from photoinhibition by promoting circulating electron flow and reducing the proton gradient across the thylakoid membrane [12]. Similarly, exogenous CaCl_2_ treatment has been observed to increase photosynthetic rate, chlorophyll content, and protective leaf enzyme activity, thereby inducing cold resistance in tomato seedlings [13]. Exogenous Ca^2+^ treatment was also found to reduce chilling damage by maintaining higher biomass accumulation of roots and shoots in maize [14] and enhancing rice seedling cold tolerance [15]. However, the role of calcium signaling in wheat cold stress tolerance regulation and the mechanisms underlying the impact of exogenous Ca^2+^ on its cold stress tolerance remains unclear.

Earlier reports revealed that cold priming can enhance wheat’s cold resistance by activating its antioxidant system and improving its photosynthesis, thus increasing the biomass of wheat plants [16]. Furthermore, cold priming significantly increased the photochemical efficiency of the photosystem, antioxidant capacity, and expression levels of cold-responsive genes in wheat [17]. However, cold priming faces challenges in its field application, and thus, alternative methods are needed to alleviate low-temperature stress occurring at the stem elongation stage. Given this context, we investigated whether Ca^2+^ shows similar functions in enhancing wheat’s tolerance to low temperatures during its stem elongation stage. In this regard, exogenous Ca^2+^ was used to determine its effect on wheat’s cold stress tolerance, and various parameters, including antioxidant capacity, endogenous Ca^2+^ content, gene expression related to Ca^2+^ signaling, and cold-responsive genes, were analyzed. These analyses aimed to explore the physiological mechanisms underlying the effect of exogenous Ca^2+^ on wheat’s cold stress tolerance.

## 2. Results

### 2.1. Effects of Exogenous Calcium on Wheat Grain Yield under Low-Temperature Stress during the Stem Elongation Stage

Compared with the control treatment (CC), low-temperature stress significantly decreased the grain yield in CL and CaL treatments, whereas an increased grain yield was observed in the CaC treatment. Notably, CaL exhibited a higher grain yield than CL, primarily due to the higher grain weight observed in CaL. Importantly, the higher grain yield observed in CaC was attributed to the higher grain weight as compared to the CC treatment (Table 1).

### 2.2. Exogenous Calcium Effect on Leaf Lethal Temperature and Photosynthetic Capacity under Low-Temperature Stress during the Stem Elongation Stage

**Figure 1:**
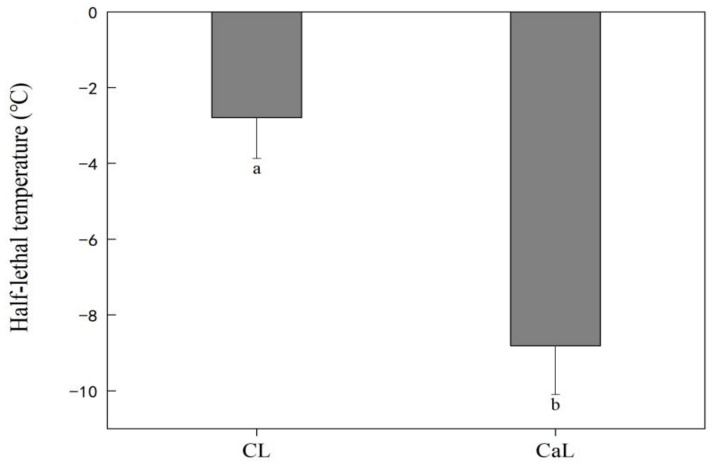
Effect of calcium on the half-lethal temperature of wheat under low-temperature stress at stem elongation stage. CL: no CaCl_2_ sprayed with low-temperature stress; and CaL: CaCl_2_ sprayed with low-temperature stress. Data are means ± SE across three biological replicates. Significant differences at *p* < 0.05 were computed, and means were separated according to Duncan’s multiple range test. The means with the same small case letters are statistically non-significant.

Compared with CL sprayed with Ca^2+^, a significantly decreased LT50 was found in CaL primarily due to the fact that Ca^2+^ can alleviate cell membrane damage under low-temperature stress (Figure 1).

**Figure 2:**
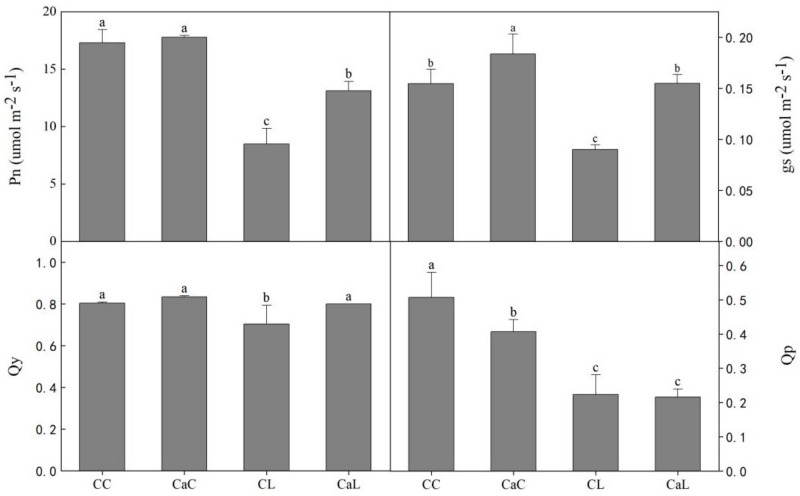
Effect of calcium on the gas exchange parameters of wheat under low-temperature stress at stem elongation stage. Pn: net photosynthetic rate; gs: stomatal conductance; Qy: quantum yield of photosystem II; qP: photochemical quenching; CC: no CaCl_2_ sprayed and no low-temperature stress; CaC: CaCl_2_ sprayed without low-temperature stress; CL: no CaCl_2_ sprayed with low-temperature stress; and CaL: CaCl_2_ sprayed with low-temperature stress. Data are means ± SE of three biological replicates. Significant differences at *p* < 0.05 were computed, and means were separated according to Duncan’s multiple range test. The means with the same small case letters are statistically non-significant.

Compared with CL, treatment with Ca^2+^ noticeably induced the net photosynthetic rate (Pn), quantum yield of photosystem II (Qy), and stomatal conductance (gs) in CaL. Except for photochemical quenching (qp), the other three parameters were increased with CaL and CaC compared to CL and CC, respectively. Due to the enhanced efficiency of the photochemical yield of PSII, the net photosynthesis increased in CaL and CaC (Figure 2).

### 2.3. Effect of Exogenous Calcium on Osmotic Regulation under Low-Temperature Stress at the Stem Elongation Stage

Compared with CL, except for free amino acids, spraying with Ca^2+^ significantly increased the contents of total sugar, fructose, and sucrose in CaL. However, no significant difference was observed for all osmotic regulators among CaC and CC (Table 2).

### 2.4. Effect of Exogenous Calcium on Cell Membrane Peroxidation Rate and Antioxidant Enzyme Activity under Low-Temperature Stress at the Stem Elongation Stage

**Figure 3:**
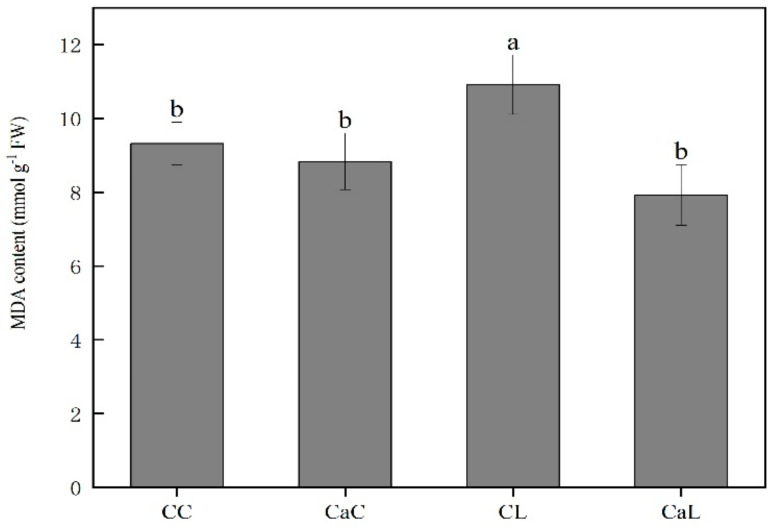
Ca^2+^ priming modifies the scavenging capacity of reactive oxygen species under the late-spring cold stress. CC: no CaCl_2_ sprayed and no low-temperature stress; CaC: CaCl_2_ sprayed without low-temperature stress; CL: no CaCl_2_ sprayed with low-temperature stress; and CaL: CaCl_2_ sprayed with low-temperature stress. Data are means ± SE of three biological replicates. Significant differences at *p* < 0.05 were computed, and means were separated according to Duncan’s multiple range test. The means with the same small case letters are statistically non-significant.

Compared with CC, the MDA content was highly increased in CL. However, the spraying with Ca^2+^ significantly reduced the MDA content in CaC and CaL, whereas there was no statistically significant difference observed between CC, CaC, and CaL (Figure 3).

Compared with CC, the treatment sprayed with Ca^2+^ enhanced the SOD, POD, APX, MDHAR, and GR activity in CaC and CaL, while no significant difference for CAT, DHAR, and GPX activity was observed in CaC and CaL (Table 3).

### 2.5. Effect of Exogenous Ca^2+^ and EGTA on Endogenous Calcium Content under Low-Temperature Stress at the Stem Elongation Stage

**Figure 4:**
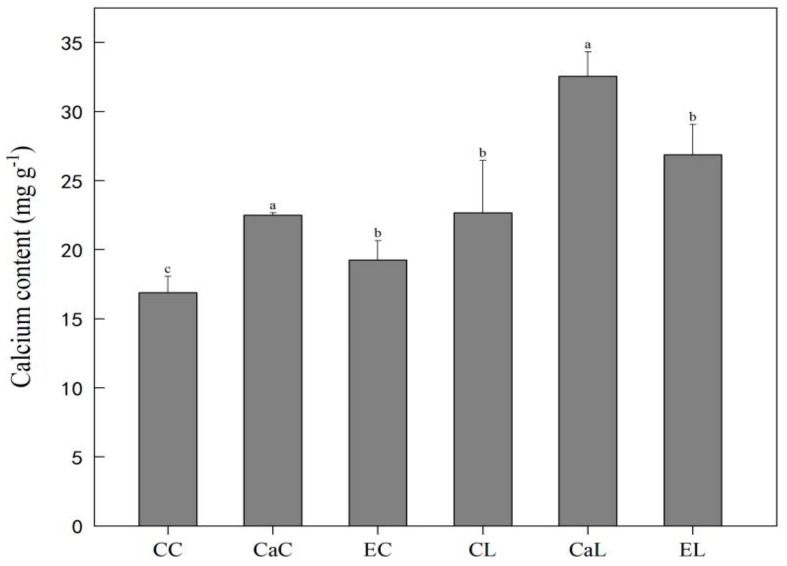
Effect of exogenous calcium and EGTA on endogenous Ca^2+^ content under low-temperature stress during stem elongation. CC: no CaCl_2_ sprayed and no low-temperature stress; CaC: CaCl_2_ sprayed without low-temperature stress; CL: no CaCl_2_ sprayed with low-temperature stress; CaL: CaCl_2_ sprayed with low-temperature stress; EC: EGTA sprayed with no low-temperature stress; and EL: EGTA sprayed with low-temperature stress. Data are means ± SE of three biological replicates. Significant differences at *p* < 0.05 were computed, and means were separated according to Duncan’s multiple range test. The means with the same small case letters are statistically non-significant.

Compared with CC, the Ca^2+^ and EGTA sprayed treatments remarkably induced endogenous calcium content in CaC, CaL, EC, and EL. Increased endogenous calcium content is associated with the exogenous Ca^2+^ treatment in CaC and CaL (Figure 4).

### 2.6. Effect of Exogenous Calcium on Gene Expression Related to Calcium Signaling and Cold-Responsive Genes under Low-Temperature Stress during the Stem Elongation Stage

**Figure 5:**
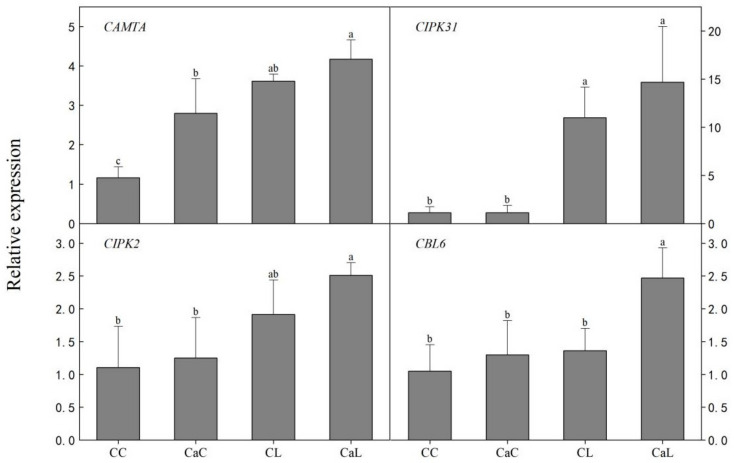
Calcium effect on gene expression of calcium signaling in wheat leaves under low-temperature stress at stem elongation. CC: no CaCl_2_ sprayed and no low-temperature stress; CaC: CaCl_2_ sprayed without low-temperature stress; CL: no CaCl_2_ sprayed with low-temperature stress; and CaL: CaCl_2_ sprayed with low-temperature stress. Data are means ± SE of three biological replicates. Significant differences at *p* < 0.05 were computed, and means were separated according to Duncan’s multiple range test. The means with the same small case letters are statistically non-significant.

Compared with CC, except CIPK31 gene, the relative expression levels of three calcium signaling genes such as CAMTA, CIPK2, and CBL6 were increased with Ca^2+^ application in CaC, whereas compared with CL, the calcium signaling genes were upregulated with Ca^2+^ spray in CaL (Figure 5).

### 2.7. Effect of Exogenous Calcium on Cold-Responsive Gene Expression under Low-Temperature Stress at Stem Elongation

**Figure 6:**
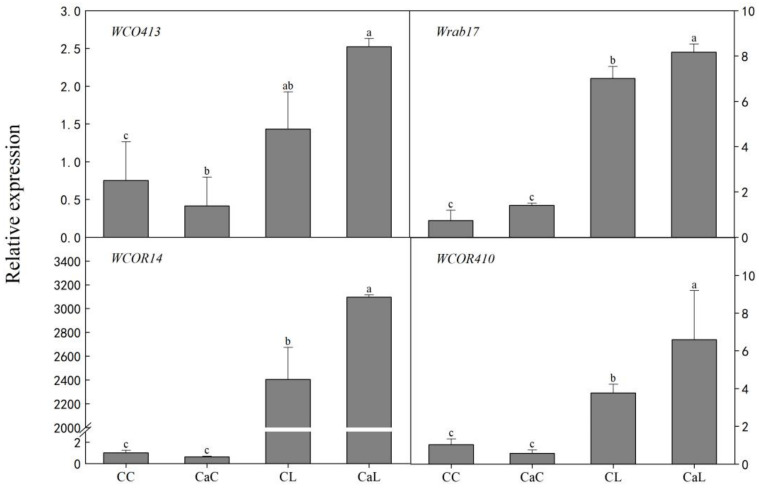
Effect of calcium on the expression of cold-responsive genes in wheat leaves under low-temperature stress at stem elongation. CC: no CaCl_2_ sprayed and no low-temperature stress; CaC: CaCl_2_ sprayed without low-temperature stress; CL: no CaCl_2_ sprayed with low-temperature stress; and CaL: CaCl_2_ sprayed with low-temperature stress. Data are means ± SE of three biological replicates. Significant differences at *p* < 0.05 were computed, and means were separated according to Duncan’s multiple range test. The means with the same small case letters are statistically non-significant.

Compared with CC, the expression levels of all measured cold-responsive genes such as WCOR413, Wrab17, WCOR14, and WCOR410 were upregulated in CL and CaL. Moreover, Ca^2+^ spraying remarkably induced the expression level in CaL compared with CL, but Ca^2+^ spraying exhibited no significant difference between CaC and CC (Figure 6).

### 2.8. Correlation Analysis

**Figure 7:**
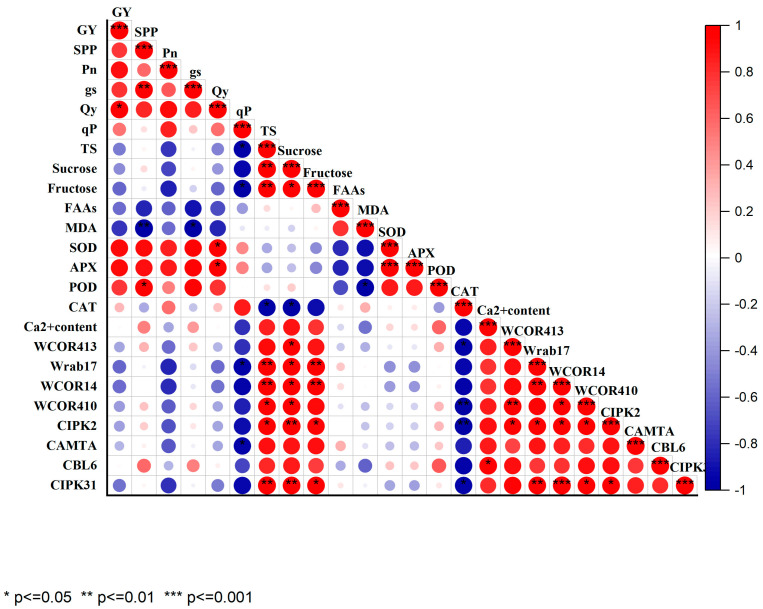
Correlation matrix of all measured indexes. GY: grain yield; SPP: spike per plant; TS: total sugar; and FAAs: free amino acids.

The correlation estimation was conducted for 24 measured traits, and the correlation coefficient scores showed a significantly strong positive correlation for 17 traits and a negative correlation for 7 traits (Figure 7). It was indicated that the yield and photosynthetic traits were positively correlated with antioxidant enzymes like SOD, POD, APX, and CAT, while a negative correlation was observed with MDA and soluble sugars. However, except for free amino acids (FAAs), the other sugars showed a strong positive correlation with calcium signaling genes and cold-responsive genes. FAAs revealed a strong negative correlation with APX, SOD, and POD, while a positive correlation with MDA. The result showed that MDA negatively correlated with *CBL6* genes, Ca^2+^ content, APX, and POD and slightly correlated with *WCOR410* gene and *WCOR413* gene. Moreover, all measured calcium signaling genes were strongly correlated with cold-responsive genes and endogenous calcium content.

## 3. Discussion

### 3.1. Exogenous Calcium Improves Plant Tolerance to Low-Temperature Stress during Stem Elongation in Wheat

Plants are highly susceptible to temperature fluctuations, and membrane fluidity is a primary characteristic that is often targeted. Low temperature degrades the cell membrane and weakens the plant tolerance ability. The damage to the chloroplast membrane negatively affects the thylakoid structure, chlorophyll content, photosynthetic enzymes, and electron transport [18]. Photosynthetic capacity is closely associated with yield formation, including under low-temperature stress [19]. The LT_50_ is an indicator of the extent of membrane damage and plant tolerance to low temperatures [20]. Plants have diverse mechanisms for tolerating cold stress that work to improve their membrane stability and enable plants to avoid any low-temperature-induced damage [21]. The current study investigated the effects of Ca^2+^ application on plant tolerance to low-temperature stress during stem elongation in wheat. Our findings showed that plants sprayed with Ca^2+^ experienced reduced cell membrane damage than non-sprayed plants under low-temperature stress.

Photosynthesis is an exceptionally sensitive process that can be influenced by even slight changes in environmental conditions, particularly in response to extreme temperatures. Maintenance of the right balance between the light energy absorbed by photosystems and the overall light energy absorbed by the plant is essential [22]. Chlorophyll fluorescence is often used to reflect a plant’s tolerance to abiotic stress [23]. Pigments play a critical role as antennae of the photosystem, trapping light energy to drive the charge separation in the reaction centers of PSII [24]. Previous studies have demonstrated that spraying Ca^2+^ can lead to enhanced maximum photochemical efficiency (Fv/Fm), enhanced PSII efficiency, and reduced relative electrolyte leakage that results in alleviating cold-induced membrane damage in rice [15]. Exogenous Ca^2+^ application was also shown to alleviate PSII photo-inhibition caused by low-temperature stress in tomatoes [13] and improve stomatal conductance, resulting in the decline of the photosynthetic rate in peanuts [25]. In line with previous studies, this study showed that wheat plants sprayed with Ca^2+^ maintained a relatively enhanced photosynthesis, likely a result of the higher stomatal conductance and reduced inhibition of photoelectron transport compared with non-sprayed plants under low-temperature stress. Our findings suggest that exogenous Ca^2+^ application may be particularly useful in improving plant tolerance to low-temperature stress, with a focus on maintaining efficient photosynthesis.

Inhibition of photosynthesis leads to an excess of electrons that can result in the formation of reactive oxygen species and membrane lipid peroxidation [26]. A direct product of membrane lipid peroxidation is malondialdehyde (MDA); thus, its concentration is an indicator of the extent of cell peroxidation [27]. In this study, low-temperature stress significantly induced the concentration of MDA when compared to the control. The lower concentration of MDA in CaL, when compared to CL, indicated lower cell membrane damage. Antioxidant systems are essential in scavenging ROS, and increasing the activity of antioxidant enzymes is crucial in the cellular defense strategy against oxidative stress [28]. Previous studies suggest that exogenous Ca^2+^ enhances ROS scavenging by increasing the activity of antioxidant enzymes and non-enzymatic antioxidants to maintain low ROS levels [29]. Consistent with this fact, our findings revealed that Ca^2+^ increased the content of all measured antioxidant enzymes under low-temperature stress when compared to non-sprayed plants. Taken together, the above results suggest that exogenous calcium application enhances plants’ defense against oxidative stress caused by low-temperature stress by improving the cellular antioxidant system and reducing oxidative damage.

The accumulation of osmolytes is another critical element of plant adaptation to low-temperature stress [30]. Sugars have been confirmed to work as excellent osmotic agents, membrane stabilizers, and antioxidant defense molecules in response to low-temperature stress [31]. Total soluble sugar was positively correlated with low-temperature tolerance [32]. Previous reports have suggested that exogenous Ca^2+^ increases osmotic regulatory substances in maize under low-temperature stress. In this study, the application of Ca^2+^ maintained higher concentrations of sugar, fructose, and sucrose, which would have decreased under low-temperature stress, in comparison to non-sprayed plants. These findings suggest that exogenous Ca^2+^ application can enhance plants’ ability to cope with low-temperature stress by improving osmolyte accumulation.

### 3.2. Exogenous Ca^2+^ Induced the Endogenous Ca^2+^ Content and Calcium Signaling Pathway

Calcium plays a crucial role as a universal messenger in various signal transduction pathways, including those responsive to abiotic stresses [33]. Previous reports suggested that Ca^2+^ pretreatment increased the endogenous Ca^2+^ concentration and upregulated *TaCaM* gene expression [34]. Furthermore, spraying Ca^2+^ on plants under cold stress significantly increased their expression level of all calcium-signaling genes [35]. It is known that the Ca^2+^ signals are triggered by low-temperature stress and the interacting protein kinases (CIPKs) receive the Ca^2+^ signal by interacting with the calcineurin B-like kinases (CBLs) that bind to Ca^2+^, thereby activating the phosphorylation activity of CIPKs [36,37]. Previous studies have indicated that rice CIPK families are upregulated by low temperatures and play a crucial role in positively regulating cold tolerance [38]. In our study, the exogenous Ca^2+^ spray increased the endogenous Ca^2+^ content. The upregulation of Ca^2+^ signaling-related genes (*CAMTA*, *CBL6*, *CIPK31*, and *CIPK2*) showed a similar tendency with the change in endogenous Ca^2+^ content. The findings suggest that exogenous calcium application induced endogenous Ca^2+^ accumulation and the upregulation of genes associated with calcium signaling pathways. Previous studies have indicated that rice CIPK families are upregulated by low temperature and play a crucial role in positively regulating cold tolerance [39]. 

In response to low-temperature stress, several genes have been identified to play crucial roles in key mechanisms [40]. Among these genes, C-repeat/DREB binding factors (CBFs) are transcription factors that have been shown to play significant roles in low-temperature stress [41]. Numerous studies have reported the existence of the CBF/DREB pathway and its ability to increase the cold tolerance of rice plants [42]. In addition to CBFs, the *WRKY19* gene has been reported to regulate the antioxidant capacity under low temperatures in *Arabidopsis* [43]. The expression of interdependent plant metabolisms facilitates functioning to counteract the deleterious effects of low-temperature stress. A previous study revealed that, after perceiving the cold signal, the expression of genes of transcription factors, i.e., *CBFs*, can be rapidly induced, and the CBF proteins then activate the cold-responsive genes by binding to the specific cis-elements of the downstream cold-responsive genes CORs, thereby enhancing the plant’s cold resistance [9,44].

COR410 is a dehydrin that is localized near the plasma membrane [45]. This protein is the terminal protein of the ICE1-CBF/DREB1-COR regulatory pathway in plant chilling injury [46]. The signal transductions and proteins that detect cold stress cause ICE genes to be activated, and as a result, ICE upregulates the expression of CBF genes. CBF proteins interact with CRT/DRE, the homeopathic component of the promoter of COR genes that activates the transduction [40]. During the cold signal transduction process, Ca^2+^ accumulation increases in the cytoplasm due to cold stress. As a result of the change in Ca^2+^ concentration, the expression of downstream genes is upregulated, indicating the positive regulation of calcium ions on the transcriptional expression of cold stress-related genes [47].

This study demonstrates that the exogenous Ca^2+^ can significantly increase the expression of cold-responsive genes (*WCOR413*, *WCOR410*, *WCOR14*, and *Wrab17*) during cold stress. This output suggests that Ca^2+^ plays a critical role in plant response to cold stress by inducing the expression of cold-responsive genes, which is consistent with previous studies [17]. Additionally, earlier studies have demonstrated that elevated expression of cold-responsive genes and antioxidant activity, cyanide-resistant respiration capacity, and molecular chaperone levels all contribute to the maintenance of cellular redox homeostasis and the safeguarding of the photosynthetic apparatus, thereby increasing wheat’s ability to withstand low temperatures. Moreover, all the results including correlation analysis suggest that Ca^2+^ can induce the expression of cold-responsive genes, leading to enhanced cold resistance and improving the overall survivability of the plant under stress conditions.

## 4. Materials and Methods

### 4.1. Experimental Design

#### 4.1.1. Experiment I

The greenhouse experiment was conducted at Nanjing Agricultural University using winter wheat, Yangmai 16, a predominant locally cultivated variety (obtained from Jiangsu Tomorrow Seed Technology Co., Ltd., Jiangsu, Nanjing, China), which was sown in plastic pots (22 cm in height and 25 cm in diameter). The plant population was reduced to seven plants per pot at the three-leaf stage. CaCl_2_ (1.5 mM) was sprayed every afternoon (6:00 p.m.) for five days during the six-leaf stage. At the stem elongation stage (the eight-leaf stage), low-temperature stress was introduced by placing pots in a growth chamber with a temperature of 2 °C/0 °C (day/night), while the control chamber was maintained at a temperature of 16 °C/12 °C (day/night). A light intensity of 500 µmol m^−2^ s^−1^ was maintained for a photoperiod of 14 h. The low-temperature stress was applied for 3 days, after which all plants were shifted to the control chamber for a seven-day recovery. Finally, all plants were grown outside until maturity. The four treatments applied were as follows: no CaCl_2_ sprayed with no low-temperature stress as a control (CC), CaCl_2_ sprayed without low-temperature stress (CaC), no CaCl_2_ sprayed with low-temperature stress (CL), and CaCl_2_ sprayed with low-temperature stress (CaL).

#### 4.1.2. Experiment II

The experiment employed the same winter wheat variety, Yangmai16, as in experiment I. Full and uniform wheat seeds were selected and disinfected with 2.5% NaClO for 10 min. The seeds were then placed evenly in wet plastic pots, on germination nets, and transferred to plastic pots (measuring 40 cm × 35 cm × 18 cm) containing Hoagland nutrient solution once they had sprouted. The seedlings were cultivated in an artificial climate chamber, with a temperature of 20 °C/16 °C (day/night, 12 h/12 h), a light intensity of approximately 500 μmol m^−2^ s^−1^, and a relative humidity of 60%. At the four-leaf stage, the seedlings were divided into three groups: one group was sprayed with 1.5 mM CaCl_2_, another with the inhibitor EGTA, and the last group was sprayed with water as a control (sprayed once every evening for five days starting at 17:00). At the six-leaf stage, half of the seedlings from the three groups were subjected to cold stress for three days in a climate chamber set at 2 °C/0 °C (day/night), while the other seedlings remained under the aforementioned growth conditions.

### 4.2. Physiological Measurements

#### 4.2.1. Leaf Half-Lethal Temperature

The uppermost flag leaves were collected for biochemical and physiological examinations. The leaf half-lethal temperature (LT50), defined as the temperature at which 50% of ions in leaves leak out, was used to evaluate the cold tolerance of wheat leaves, with slight modifications made to the measurement method described by [48]. Fresh leaves were harvested after exposure to low-temperature cold stress and cut into 1.5 cm lengths. These leaf segments were then wrapped in a wet cotton cloth and incubated overnight at 4 °C. After that, the sample was exposed to various freezing temperatures (0 °C, −3 °C, −6 °C, −9 °C, −12 °C, and −15 °C) in a freezing bath (CDN-1007020F, Southeast Co., Ltd., Ningbo, China) before being thawed overnight at 4 °C. Thereafter, 15 mL of deionized water was added, and the leaves were extracted at room temperature for 12 h, following which, the electrolytic conductivity of the leachate was measured before and after boiling the leaves for 15 min with a conductivity meter at 20 °C (DDS-307A, LEX Instruments Co., Ltd., Shanghai, China).

#### 4.2.2. Leaf Photosynthesis and Chlorophyll Fluorescence

Leaf photosynthetic rate (Pn) and stomatal conductance (gs) were measured using a Licor-6800 portable photosynthetic instrument (Li-COR Biosciences, Lincoln, NE, USA) between 9:00 and 11:00 a.m. The instrument was configured with a red and blue light source, with the CO_2_ concentration maintained at 400 µmol mol^−1^ and photosynthetically active radiation set at 1000 µmol m^−2^ s^−1^. Chlorophyll fluorescence characteristics were assessed using the Fluor Pen FP100 (Beijing Eketai Ecological Technology Co., LTD, Beijing, China) following the manufacturer’s instructions [49]. The minimum fluorescence (Fo) and maximal fluorescence (Fm), which were dark-adapted, variable fluorescence (Fv) and light-adapted fluorescence, such as steady-state fluorescence (Fs), and maximal fluorescence (Fm′) were recorded. All the fluorescence parameters were calculated using the method reported in [50]. Accordingly, the maximum photochemical efficiency of PSII (Fv/Fm), Fv/Fm = (Fm − Fo)/Fm; the actual quantum yield of PSII (ΦPSII), (ΦPSII) = (Fm′ − Fo)/Fm; and the non-photochemical quenching (NPQ), NPQ = (Fm − Fm′)/Fm, were calculated.

#### 4.2.3. Osmolyte Content

A total of 0.05 g of dry leaves were weighed and 14 mL of 80% ethanol was added to it three times in a 15 mL centrifuge tube, which was then shaken vigorously. The mixture was heated at 80 °C for 30 min in a water bath and centrifuged at 3000 rpm for 10 min. The supernatant obtained was combined, and the solution was diluted to a final volume of 15 mL. This extract was utilized for determining the total sugar content using the anthrone method [17] and the sucrose content using the resorcinol method according to [51].

#### 4.2.4. Cell Membrane Damage and Antioxidant Enzyme Activities

The last fully expanded leaves were utilized for extracting cell membrane damage and antioxidant enzyme activities. Approximately 0.5 g of fresh leaves was homogenized in 5 mL of precooled buffer (50 mM HEPES buffer, pH 7.8) followed by centrifugation at 10,000 rpm for 20 min at 4 °C. The resulting supernatant was collected to determine antioxidant enzyme activity and MDA content, which was measured according to the method described by [52]. The reaction solution, containing trichloroacetic acid, thiobarbituric acid mixture, and 2 mL of the extract, was boiled in a water bath for 20 min. This solution was then centrifuged at 4000 rpm for 10 min, and the supernatant was measured at 450 nm, 532 nm, and 600 nm using a visible light spectrophotometer (UV-1780, Shimadzu (Suzhou) Instruments Manufacturing Co., Ltd., Suzhou, China). Enzyme activity, including that of guaiacol peroxidase (POD), catalase (CAT), superoxide dismutase (SOD), ascorbate peroxidase (APX), glutathione reductase (GR), dehydroascorbate reductase (DHAR), and monodehydroascorbate reductase (MDHAR), was determined according to the methods described by [21]. The total soluble sugar content was measured following the recommended methods outlined by [6].

#### 4.2.5. Endogenous Calcium Content

The Ca^2+^ content in leaves was determined using a Calcium Colorimetric Assay Kit (Biyuntian, Nanjing, China). Fresh leaf samples (50 mg) were mixed with 500 μL of the cracking solution. Samples were fully cracked and then centrifuged at 10,000 rpm for 3–5 min at 4 °C, and the obtained supernatant was utilized for testing. Following the instructions provided with the kit, the OD value at 575 nm was measured using a Microplate Reader (BioTek Cytation 3, Nanjing, China) [53].

#### 4.2.6. Gene Expression

In order to measure gene expressions, total RNA extraction, cDNA synthesis, and quantitative real-time PCR (qRT-PCR) were carried out as described by [53]. In Table 4, all primer sequences used in qRT-PCR are mentioned. Using the Actin gene as a reference gene, the relative expression levels of the various genes were determined using the 2^−ΔΔCt^ method.

### 4.3. Statistical Analysis

An analysis of variance (ANOVA) was conducted using the statistical software SPSS version 25.0 (SPSS Inc., Chicago, IL, USA). The means were separated using Duncan’s multiple range testing at a significance level of *p* < 0.05 using SigmaPlot 12.5 (Systat Software Inc., San Jose, CA, USA). The correlations matrix was plotted according to Pearson’s method by using Origin 2023 (OriginLab Inc., Northampton, MA, USA).

## 5. Conclusions

In conclusion, this study examined how exogenous Ca^2+^ affected the expression of genes, the physiological and biochemical responses, and the stress tolerance of wheat when exposed to low temperatures. The results show that Ca^2+^ induces the expression of Ca^2+^ signaling pathway genes and genes involved in cold stress responses of the plant. The upregulated expression of calcium-signaling genes (*CAMTA*, *CBL6*, *CIPK31,* and *CIPK2*) and cold-responsive genes (*WCOR413*, *WCOR410*, *WCOR14*, and *Wrab17*) maintained cellular redox homeostasis and a higher photosynthesis rate, and ultimately enhanced low-temperature stress tolerance in wheat. This study establishes the regulatory mechanisms of calcium for low-temperature stress tolerance in wheat, which provides a new avenue for future research on the adaptive mechanisms of plants to environmental stress.

## Figures and Tables

**Table 1:** Effect of calcium on wheat yield and its components under low-temperature stress during stem elongation.

Treatment	Spikes	Kernels per Spike	Thousand Grain Weight (g)	Yield (g)
CC	13.00 ^ab^	51.67 ^b^	50.35 ^b^	33.03 ^b^
CaC	13.25 ^ab^	57.13 ^a^	54.60 ^a^	39.98 ^a^
CL	11.25 ^b^	41.53 ^d^	48.35 ^b^	20.76 ^c^
CaL	14.25 ^a^	46.00 ^c^	52.71 ^a^	33.00 ^b^

**Note:** CC: no CaCl_2_ sprayed and no low-temperature stress; CaC: CaCl_2_ sprayed without low-temperature stress; CL: no CaCl_2_ sprayed with low-temperature stress; and CaL: CaCl_2_ sprayed with low-temperature stress. Significant differences at *p* < 0.05 were computed, and means were separated according to Duncan’s multiple range test. The means with the same small case letters are statistically non-significant.

**Table 2:** Effect of calcium on osmotic regulators under low-temperature stress during stem elongation.

Treatment	Total Sugar (mg g^−1^ DW)	Fructose (mg g^−1^ DW)	Sucrose (mg g^−1^ DW)	Free Amino Acids (mg g^−1^ DW)
CC	35.96 ^c^	21.23 ^c^	4.79 ^c^	45.68 ^a^
CaC	46.45 ^c^	30.34 ^c^	9.37 ^c^	46.98 ^a^
CL	108.53 ^b^	83.58 ^ab^	47.20 ^b^	48.14 ^a^
CaL	133.49 ^a^	91.23 ^a^	71.81 ^a^	45.73 ^a^

**Note:** CC refers to blank control, CaC refers to only CaCl_2_ spraying, CL refers to direct low-temperature treatment, and CaL refers to low-temperature treatment after CaCl_2_ spraying. Significant differences at *p* < 0.05 were computed, and means were separated according to Duncan’s multiple range test. The means with the same small case letters are statistically non-significant.

**Table 3:** Effect of calcium on the activity of antioxidant enzymes in wheat leaves under low-temperature stress at stem elongation.

Enzymes	CC	CaC	CL	CaL
SOD (U mg^−1^ protein)	4.01 ^c^	10.34 ^a^	6.56 ^b^	11.37 ^a^
POD (umol mg^−1^ min^−1^ protein)	2.21 ^c^	3.06 ^a^	2.59 ^b^	3.26 ^a^
CAT (umol mg^−1^ min^−1^ protein)	1.70 ^ab^	1.79 ^ab^	1.29 ^b^	2.09 ^a^
APX (umol mg^−1^ min^−1^ protein)	0.55 ^b^	1.14 ^a^	0.23 ^b^	1.15 ^a^
MDHAR (umol mg^−1^ min^−1^ protein)	1.16 ^b^	1.64 ^a^	0.79 ^c^	1.69 ^a^
DHAR (umol mg^−1^ min^−1^ protein)	7.10 ^b^	10.73 ^b^	8.02 ^b^	17.91 ^a^
GPX (umol mg^−1^ min^−1^ protein)	0.90 ^bc^	1.46 ^ab^	0.82 ^c^	1.86 ^a^
GR (umol mg^−1^ min^−1^ protein)	3.89 ^b^	5.42 ^a^	4.20 ^b^	5.99 ^a^

**Note:** CC: no CaCl_2_ sprayed and no low-temperature stress; CaC: CaCl_2_ sprayed without low-temperature stress; CL: no CaCl_2_ sprayed with low-temperature stress; and CaL: CaCl_2_ sprayed with low-temperature stress. Significant differences at *p* < 0.05 were computed, and means were separated according to Duncan’s multiple range test. The means with the same small case letters are statistically non-significant.

**Table 4:** Primer used in the q-PCR analysis of genes expressed in wheat leaves.

Gene Name	Forward Primer Sequence	Reverse Primer Sequence
CAMTA	ATGGGAGTTGGGCGGTGATG	CATGTTCTCTTCGCCATCAC
CBL2	AGACGAGCAAGAAGGAGAGC	CTGAAGCATTTGGGTGAAAC
CBL4	TCAAGAAGAACCCGGCATCAC	CCGAATGCATCACAAAGCTCGG
CBL6	GACATACCAAATCGTCCCAAG	ATCACACCATCATCAACCACAG
CIPK2	TGCATTCCCCTAGTGATGTCTG	CCATACACAAACGCACTGTCC
CIPK29	ACGCGCAAGAAGGTCCACTT	ACACGAGCTGGCGGAAGTAA
CIPK31	CAGCCCACTGTGGAAGAGC	TTACAACAATCGGCCTTTCGC
MAPK2C	GATTGTAAGCTCAAAATATGTG	ACATAGTTCAGGTGCTCGGTA
MAPKflrs	GCAAACTGTGACCTAAAA	ACAGAAGCTCTGGTGCC
ICE1	CAACAAGGTCGTAGGAGATG	CCAATCAGCATAAGAAAACG
ICE4	CAAGGGCAAGAAGAAGGG	GCGTCACCAAGGATTGAA
WCOR14	CGACCACCAGACCCAGACC	CGAGCGGCGAGGAAACAC
WCOR15	CTGGTTAGTCGTCCTCTGA	CCTTCTTCAACTCGTCGG
WCOR18	GTGGACGCACTGGGTGGTC	ACCTTGTTGCCGAGGCTGA
WCOR410	CCTCCTCGGCAACCTCCTC	TCTTGACCTCGGGCTCTTCC
WCOR413	GACAAGACGAACTGGAAGA	ACAACAACGAACGCAATC
Wrab17	GATGCCACCAAGGAGAAGTC	CTCACTTGTCTCCTCCCATC
Actin	ACAGTGTCTGGATCGGTGGC	GTGGACAATGCCGGGACCAG

## Data Availability

Both the raw data and analyzed data sets are available from the corresponding author upon reasonable request.
